# An Environmental Monitoring System for Managing Spatiotemporal Sensor Data over Sensor Networks

**DOI:** 10.3390/s120403997

**Published:** 2012-03-27

**Authors:** Su Wook Ha, Yang Koo Lee, Thi Hong Nhan Vu, Young Jin Jung, Keun Ho Ryu

**Affiliations:** 1 Robot/Cognitive System Research Department, Electronics & Telecommunication Research Institute, 218 Gajeong-ro, Yuseong-gu, Daejeon 305-700, Korea; E-Mail: suwook.ha@etri.re.kr; 2 Database/Bioinformatics Laboratory, Chungbuk National University, 12 Gaeshindong, Cheongju, Chungbuk 361-763, Korea; 3 Faculty of Information Technology, University of Engineering and Technology, VNU, 144 Xuan Thuy, Cau Giay, Hanoi 10000, Vietnam; E-Mail: vthnhan@vnu.edu.vn; 4 Department of Cyber Environment Development, Supercomputing Center, Korea Institute of Science and Technology Information, 245 Daehak-ro, Yuseong-gu, Daejeon 305-806, Korea; E-Mail: yjjung@kisti.re.kr

**Keywords:** wireless sensor network, senor data stream, spatiotemporal continuous query processing, data stream management system

## Abstract

In a wireless sensor network, sensors collect data about natural phenomena and transmit them to a server in real-time. Many studies have been conducted focusing on the processing of continuous queries in an approximate form. However, this approach is difficult to apply to environmental applications which require the correct data to be stored. In this paper, we propose a weather monitoring system for handling and storing the sensor data stream in real-time in order to support continuous spatial and/or temporal queries. In our system, we exploit two time-based insertion methods to store the sensor data stream and reduce the number of managed tuples, without losing any of the raw data which are useful for queries, by using the sensors' temporal attributes. In addition, we offer a method for reducing the cost of the join operations used in processing spatiotemporal queries by filtering out a list of irrelevant sensors from query range before making a join operation. In the results of the performance evaluation, the number of tuples obtained from the data stream is reduced by about 30% in comparison to a naïve approach, thereby decreasing the query execution time.

## 1. Introduction

Wireless Sensor Networks (WSNs) consist of a large number of sensors located in the physical world that collect and communicate data continuously [1–3]. Modern hardware technologies make it possible to gather data by using cheap and small sensor devices (e.g., smart dust and RFIDs). These sensors collect data about natural phenomena such as the temperature, light, sound, and pressure and then transmit them to a server in real-time. They are widely utilized in geophysical monitoring, movement tracking, and medical monitoring [4–6]. Sensor networks have been deployed successfully in applications for environmental monitoring (e.g., disaster management, air pollution monitoring, ecology monitoring, and early-warning system). Sensors are spatially scattered on a large scale to continuously measure information from their surrounding environment. Gathered data are used for statistical analysis and long-term decision making [7]. So far many studies in the field of sensor networks have been concerned with how to design good environmental sensor networks. Problems concerning sensing, communication, and computing are explored [8]. Issues related to in-network processing, stream processing technology or integrated architecture under two or three layer system architectures are also addressed [9–12]. However, there are still a lot of issues arising from the application level that have not been fully addressed by those previous works yet, such as how to efficiently and sensibly store and manipulate a huge amount of current streaming data as well as historic data.

In this paper we develop a weather monitoring system. Oftentimes data are autonomously and regularly sampled from sensor nodes. In other words, sampling time intervals are pre-specified. If that interval is too large, useful information may be missed. However if it is too small, information from the environment is almost constant. If this is the case, data processing would be time-consuming. We would waste a significant amount of precious storage capacity due to data replication. Data can be either recorded in *in-situ* storage or transmitted to an application server through one or more powerful sensor nodes (i.e. base stations). The server in this case should accumulate and manage all the data streaming in in an optimal manner so that it is able to support even complex dynamic queries like spatial and/or temporal queries. Such queries cannot be dealt with by the same methods used for one-time queries on static data in traditional database systems. Currently a good mechanism for processing queries over streaming sensor data is still a crucial demand. To address these problems, sensor network technology needs to be firstly extended to monitor widely distributed sensing devices without human interference. Secondly, it must be able to support the user's decision-making by analyzing the gathered data from the area covered by sensor network. Besides the constructed system should be capable of answering the following continuous queries:

Query 1: Return the temperature in *State_A*, every 10 minutes.
Query 2: Return the temperature of the last 5 days in *State_B*, every 10 minutes.

To obtain the results of these queries, the application system has to perform join operations relating to spatial, temporal, or spatiotemporal conditions. Accordingly, it is necessary to find a solution to the problem of efficiently processing the complex queries pertaining to spatial and/or temporal join operations. In addition, a sensor query sometimes requires an answer for a long interval, as in the following example:

Query 3: Return the average temperature measured by all sensors last month.

This type of historical query is mainly required for periodic analysis or statistics of the data stream. The data measured by the sensors in some applications, weather monitoring for example, rarely change over a certain time-point and all of the measured data need to be stored.

In this study, we present a weather monitoring system based on the existing temporal and spatial approaches, in order to support spatiotemporal queries and store sensor data. In our system, we introduce two insertion methods called Time-Segment Insertion (TSI) and Time-Point Insertion (TPI). These methods save storage space without any loss of the raw data necessary for queries using the sensors' temporal attributes. In the TSI method, the incoming data stream is stored in the form of a time-segment that records two timestamps corresponding to the times at which the value of the item is changed. In the case of the TPI, each tuple (*i.e.*, sampled record) is attached only to a time-point and tuple insertion is only performed when its value changes. These methods can help to store the incoming stream without any loss of information, while maintaining the history of the data in memory as long as possible, because only one tuple needs to be kept in memory when it duplicates the values acquired over some interval.

Additionally, the spatial information about the sensors is kept resident in memory with the assistance of a fixed grid that identifies the sensors' locations. This method is capable of reducing the cost of a join operation, due to the filtering out of the list of irrelevant sensors from the query range before making the join operation. Besides, the historical data stream evicted from memory is stored to disk. Although the cost of disk access is high, this operation rarely occurs, because the stored data stream is already reduced in size while in memory.

In our experiments, we compare the TSI and TPI methods with the Non-Temporal Insertion (NTI) method as a naïve method which is used in most data stream systems and has no consideration of the time representation. Moreover, we evaluate the performance of these methods with the use of factors such as the number of tuples, average insert and query execution time, along with the number of sensor readings obtained from the incoming data stream. The results show that the proposed methods are better than the NTI method in terms of the data storage as well as the query execution time.

The remainder of this paper is organized as follows: in Section 2, we briefly review related work. Section 3 introduces how the data stream pertaining to the spatial and temporal attributes is managed to tackle spatiotemporal queries. The design of the system architecture for the implementation of the proposed approach is introduced in Section 4. In Section 5, we present the system implementation and a running example illustrating a weather monitoring. The performance evaluation and analysis are presented in Section 6. Finally, we conclude this paper and describe the directions of our future work in Section 7.

## 2. Related Work

Before any system is designed and installed, a detailed understanding of its physical environment and deployment is required. Design of environmental sensor networks has been approached by many researchers. Research areas including sensing, communication and computing have been examined extensively [8]. Work in [7] designed a WSN for habitat monitoring. The requirements of environmental monitoring in the context of two wildlife habitats: Great Duck Island and James Reserve were examined first. Based on the requirements of the researchers studying these habitats, a sensor network architecture for this class of application was proposed, and the hardware design: sensor platform, enclosure design, and sensor calibration was then discussed.

Environmental Observation and Forecasting Systems (EOFS), are a new class of large-scale distributed systems designed to monitor, model, and forecast wide-area physical processes such as river systems. EOFS have strong social relevance in areas such as education, transportation, agriculture, natural resource planning and disaster response. CORIE [13], FLOODNET [14], GLACSWEB [15] record environmental information, such as temperature, salinity, water levels, and flow velocities, and transmit this information to a centralized compute. CORIE uses sensor stations in the Columbia River to get various types of environmental data. FLOODNET, an example of pervasive computing, tries to check a functional floodplain condition at a particular location by using wireless sensor network with an automated adaptive sampling approach. The GLACSWEB monitors the behavior of ice caps and glaciers for understanding the Earth's climate. The sensor information is used to drive 2-D and 3-D fine-grain environmental models. The output of the models has been used for a variety of purposes, including online control of vessels, marine search and rescue, and ecosystem research and management. Our research does not focus on hardware design but rather on the application layer of the system.

A very recent work on design of sensor data processing steps for an air pollution monitoring system has been presented in [16]. This research focuses on visualizing collected data and experts would assess environment change based on the visualized patterns. This system does not provide a mechanism for answering queries that are often asked by users without expert knowledge.

Query processing and data management in sensor network has been done by many researchers. COUGAR [17] generates an efficient query plan aimed at minimizing resource usage within the network at a central query optimizer. TinyDB [18] uses an acquisitional query processing approach whereby data is requested from sensors depending on the specific query posed to the network. BBQ [19] improves over TinyDB by constructing data models of the sensed data using statistical modeling techniques. Although these approaches focus on continuous query processing for data at the current time, they do not take the storage management for historical queries into account, which would be useful for subsequent statistical reports and decision making.

To process data streams, Borealis in [11] works on the integration framework between sensor network and DSMS (Data Stream Management System) in which energy efficiency is considered. They provide integrated system architecture based on DSMS system and sensor network to support the query processing mechanism, but they discuss the transactional data stream rather than the sensor data and do not consider characteristics of sensor network. StonesDB [12] assumes that sensors have large local storage (e.g., NAND) and store data to this storage. StonesDB is a 2-tiered integration system which consists of a low-tier including a battery-powered and resource-constrained sensor network, and a higher-tier including power-rich proxies. Our approach concentrates more on how to optimize users' queries in which include spatiotemporal predicates in application layer while StonesDB mainly focuses on the whole system architecture and a conceptual view for implementing systems.

Another research area related to ours is data indexing. Issues concerning indexing have been extensively studied in the context of spatial databases. Most cases are focused on R-tree based indexes [20] which are adaptable to ununiformed data distributions such as Gaussian and skewed. Besides, in the case of uniform distributions like sensor networks it is preferable to use grid-based indexes like fixed grid and grid file for the propagation of the sensor network, because grids can easily maintain the index structure and access the pages directly in environments in which data rarely cause overflow in pages and updates [21]. In the context of temporal databases [22], a temporal foundation for stream algebra is attempted, which makes a distinction between the logical and physical operator levels. Transformation rules are provided between a logical level that refers to a query specification and a physical level that covers implementation issues.

## 3. Spatiotemporal Sensor Data Stream Management

### 3.1. Time-Based Insertion Methods to Eliminate Duplicate Data

The sensor data stream that is collected at regular time-points is a temporally ordered dataset. Especially, the values of an environmental data stream, which is composed of the temperature, humidity and illumination, for example, rarely change for some specific time-point and the storage of the correct values is required. Since sensors generate massive volumes of data, it is impossible to store all the data to disk and then query them. Therefore, the incoming stream is mainly dealt with in memory. In this paper, we introduce two methods called Time-Segment Insertion (TSI) and Time-Point Insertion (TPI). These methods store the correct incoming data stream using the temporal attribute. TSI stores the incoming stream as tuples associated with the time-segment and TPI stores the incoming stream as tuples associated with a time-point that takes the same values for some timestamp.

#### 3.1.1. Time-Segment Insertion Algorithm

**Figure 1.**
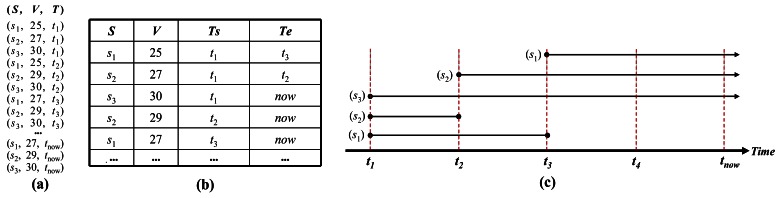
Processing of TSI method (**a**) incoming data stream; (**b**) storage of table in memory; (**c**) time-segments representing the lifetimes of the items.

In the TSI method, before inserting the data into memory, we compare the new value of the data with the previous value and if the value has changed, we update the data that are attached to the timestamp at which the value is changed. If the data has the same value as the previous one, the tuple's timestamp of this data in memory is maintained as “*now*”. Let the datasets of the incoming stream be denoted as <*S, V, T*> where *S* is the sensor identifier, *V* determines the measured value, and *T* is the timestamp. Additionally, let the table be <*S, V, Ts, Te*>, where *Ts* and *Te* are the start time and end time of the new value. Here, *Te* is “*now*” when the item's value is valid until the present time. *Te* will be updated with the timestamp at which the data is changed and, at the same time, a new tuple will be inserted into the table, whose timestamp is *Ts*. Figure 1 shows an example of the representation of the incoming data stream. Figure 1(a) illustrates the processing of the TSI method and Figure 1(b) represents the table whose tuples store the values of the data stream after they pass through the memory. Initially, at timestamp *t*_1_, the items *s*_1_, *s*_2_, *s*_3_ are inserted into the tables, and each one is associated with a memory value whose *Ts* is *t*_1_ and *Te* is “*now*”. Next, at timestamp *t*_2_, the end-time's value “*now*” of *s*_1_ is maintained; because the sampled value of *s*_1_ is still the same, *i.e.*, 25. However, the *Te* of *s*_2_ at timestamp *t*_1_ is updated with *t*_2_, since *s*_2_ is sampled and its value has been changed from 25 to 27, so the new value 27 should be stored by inserting a new tuple into the table and giving the field *Ts* of this tuple the timestamp *t*_2_. In the same manner, at timestamp *t*_3_, the tuple of *s*_1_ is updated. Finally, when all the items of Figure 1(a) are inserted, the tuples in the table are represented as shown in Figure 1(b). We realize that in the case of *s*_3_, only one tuple is kept because the sampled values of *s*_3_ are constant from *t*_1_ to *t*_now_. Figure 1(c) displays the timestamps of *Ts* and *Te* over the whole incoming stream.

**Figure 2.**
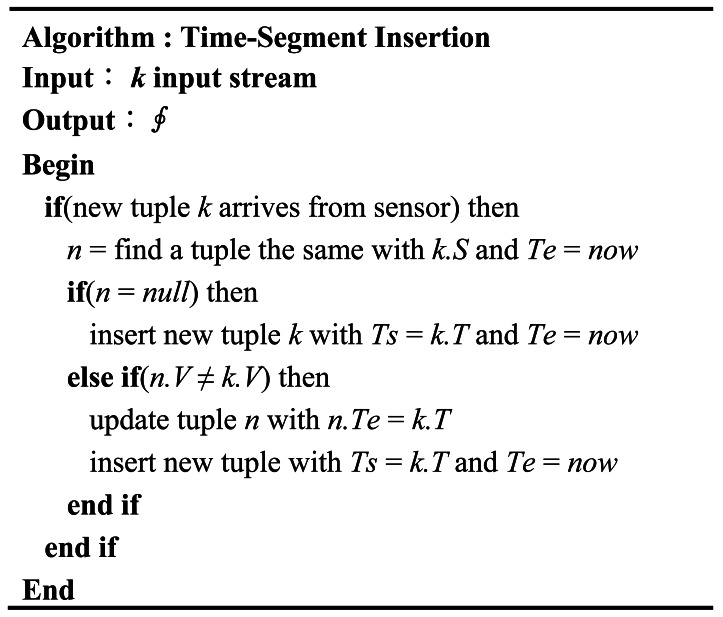
Time-Segment Insertion Algorithm.

The TSI algorithm, shown in Figure 2, only stores those items whose measured values are found to have changed after comparing the incoming stream with that stored in memory. If the data stream has the same values as before, we discard the data without storing it in memory. The disadvantage of the TSI method is that it incurs additional overhead due to the update operation. The reason is that TSI has to update the lifetime of the tuple in memory with the time interval when the values are different from the previously stored values. On the other hand, if the sensor frequently transmits the same values for some timestamps, the operation to store the new values is not required. Therefore, the overhead caused by the update operation may be reduced. Moreover, the TSI method has the advantage that it can keep the values of the data without any loss of data and store the data stream in memory as much as possible.

#### 3.1.2. Time-Point Insertion Algorithm

**Figure 3.**
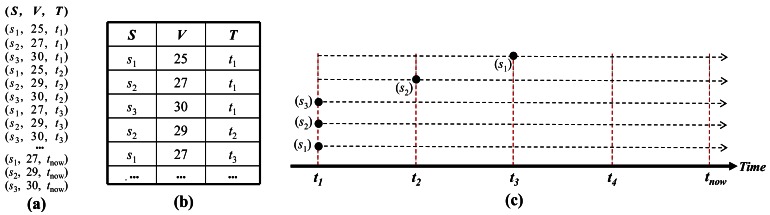
Processing of TPI method (**a**) incoming data stream; (**b**) storage of table in memory; (**c**) time-points representing the measured timestamp.

Generally, an update operation has a higher cost than an insert operation, because of the necessity of finding the corresponding tuple's identifier and changing the time-interval of the tuple. In the case of TSI, the higher the frequency with which the values of the incoming stream change, the higher will be the update cost in comparison with the insert cost. The update cost is reduced only when some tuples are discarded. The TPI method stores the incoming data stream by attaching the tuples to a time-point, thereby solving the problem of the overhead associated with updating in TSI. Figure 3 illustrates the processing sequence used by the TPI method to store the incoming stream in the form of tuples with a time-point. As in the case of the TSI method, the TPI method stores a time-point when *s*_1_, *s*_2_, *s*_3_ have arrived at timestamp *t*_1_, and at a subsequent timestamp, if their values change in the incoming data stream, a new tuple with changed values and a new time-point is inserted. Until timestamp *t*_now_, the unchanged data values are discarded.

**Figure 4.**
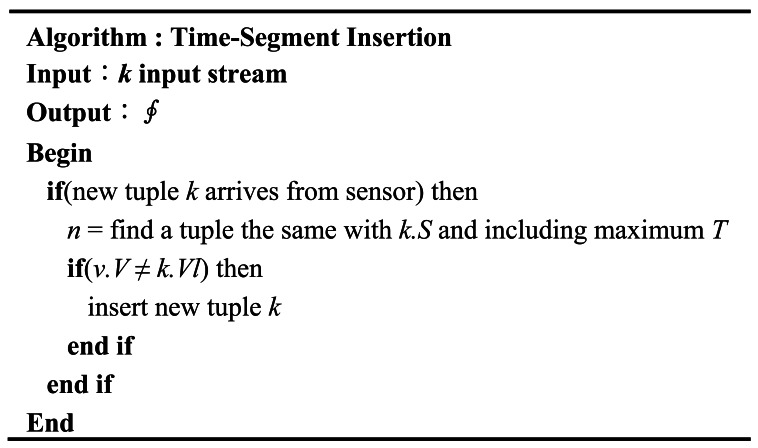
Time-Point Insertion Algorithm.

Figure 4 represents the pseudo-code of the TPI algorithm. This algorithm is the same as the TSI algorithm as regards the comparison of the incoming data stream with the previously received one. However, the TPI method only requires that the changed data be stored with the time-point and, therefore, does not need an additional update operation. Consequently, the TPI method has the advantage of reducing the total storage cost of the incoming stream.

### 3.2. Location-Based Spatial Filtering

Generally, sensors are fixed at a location until their battery is depleted, so their spatial attributes are static. The spatial attributes of sensors are managed by the server and it refers to them when a query demands spatial information (See Definition 1):

**Definition 1.** Spatial range is a set of the coordinate including the latitude and the longitude of sensor nodes distributed on a wide spatial area. The spatial range is represented by S_range_ = <x_1_, y_1_, x_2_, y_2_> and this can be referred to a specific group name, for example, S_range_ = State_A.

For example, in the case of a new incoming data stream, the server selects the data satisfying the query predicate from the incoming data stream. If the query pertains to the spatial attribute, the query processor executes it along with the spatial attributes in memory. Consider the example Query 1 in Section 1. In this case, the query processor searches the distributed sensors in State A. However, the problem is that the query processor searches all the spatial attributes of the sensors for the ids satisfying the query's predicates. If the query processor can refer to an index scheme for the list of sensors, the cost of query processing is reduced, because the search will be performed within a small spatial scope.

**Figure 5.**
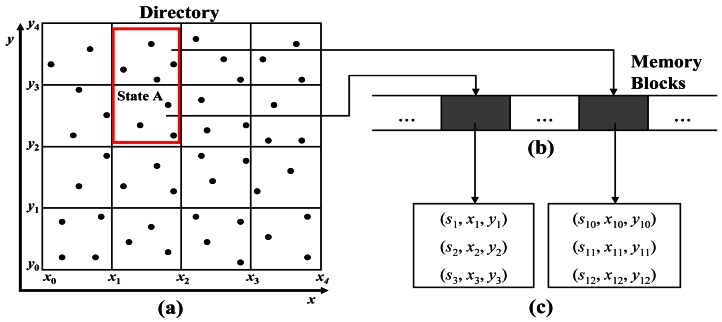
Spatial mapping for searching for the locations of sensors. (**a**) directory of fixed grid for location mapping; (**b**) memory blocks referenced by the directory; (**c**) item set in memory blocks.

Therefore, we apply a fixed grid in processing continuous queries pertaining to spatial attributes. The fixed grid can easily maintain the index structure and access the pages directly in some environments like sensor networks in which data rarely causes an overflow in pages and updates. Figure 5 illustrates the spatial mapping used for searching for the locations of sensors. Figure 5(a) shows that all the locations of the sensors are mapped into the fixed grid. Figure 5(b) presents the memory blocks that are pointed to by the directory. A memory block stores the list of sensors, which are located in each cell area. Figure 5(c) depicts the list of sensors, which are stored in the memory block.

Because the fixed grid only requires sufficient memory space to store the pointers used for referring to the memory block, this structure uses only a small amount of additional memory. Moreover, the fixed grid has the advantage that it can filter the list of sensors in a query predicate in advance. In particular, for a join operation relating to the temporal predicate and spatial predicate in spatiotemporal historical queries, this structure can reduce the cost of the join operation.

### 3.3. Spatiotemporal Query Processing

Queries on a sensor data stream mostly involve temporal, spatial, and spatiotemporal attributes. The problem is that a complex query such as a spatiotemporal query requires a join operation between spatial and temporal intervals. In this case, it is inefficient to join all the locations with the time-point. The solution mentioned in the previous section can reduce the cost of query processing by filtering the tuples and location before making the join operation:

**Definition 2.** Spatiotemporal range is defined by the spatial location and a temporal range. Spatial range is represented by S_range_ = <x_1_, y_1_, x_2_, y_2_> and temporal range is defined as T_range_ = <t_s_, t_e_> with 0 ≤ t_s_ ≤ t_e_.

**Figure 6.**
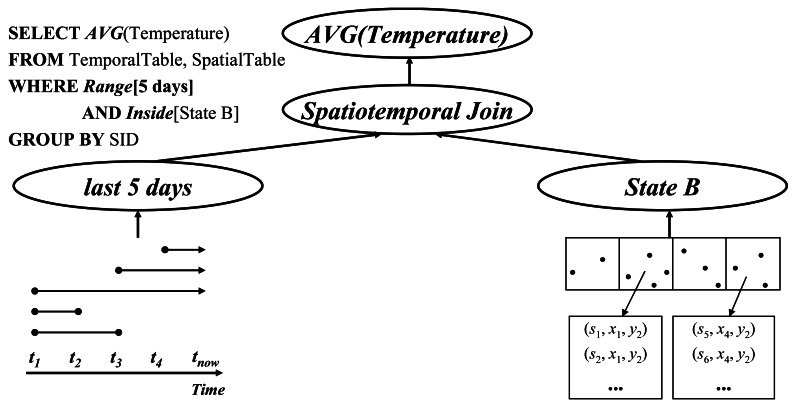
Spatiotemporal historical query join operation.

An example query involving a spatiotemporal join (e.g. Query 2 in Section 1) is represented by the SQL in Figure 6. The join step of the query is as follows: The first step is identifying those cells that are limited by the spatial scope in the query predicate, and get the list of sensor ids stored in the memory blocks referred to by the directory. Then, select all of the tuples belonging to the last 5 minutes from the data stream. Finally, execute the join operation with the sensor ids between the two results.

### 3.4. Historical Data Stream Management

The data stream in memory can be used to obtain the answer to a historical query on the recent data stream for a short time-point. However, when we need the query result to analyze data or to retrieve statistical data, we have to search the data from some time ago. Consider the example of Query 3 in Section 1. To answer this query, we need the archive of the data since last month, but the memory cannot be used to keep the data for such a long time. Besides, in order to store the recent data stream in the memory, the old data has to be discarded from memory or stored on the disk while storing the new incoming data stream. Also, if the data which cannot be kept in memory anymore are discarded completely, the accuracy of the data could be decreased, and if all of the old data are stored to the disk, the resulting disk accesses lead to a certain system overhead.

In the proposed TSI and TPI methods, we reduced the number of items stored in memory in the case where the data from the incoming data stream which are to be removed have the same values. This means that the number of items stored on disk is also less than the number of incoming items. Consequently, the number of disk accesses is reduced. Therefore, the proposed methods are suitable for a temporal database. Because the data stream along with the timestamps can be kept on disk without any change of the data format, the data stream managed by a temporal database still preserves the order of the data sequence. This is the reason why we can apply the temporal database without any modification. Moreover, such storage of data ensures the accuracy of long-interval historical queries.

## 4. System Architecture

A variety of data stream management systems have been introduced in previous studies of data streams. They generally present the system architectures required to support the processing of continuous queries on the data stream in real-time. The system proposed in this paper modifies the existing architecture in order to manage historical data, which are necessary to deal with spatiotemporal queries continuously.

**Figure 7.**
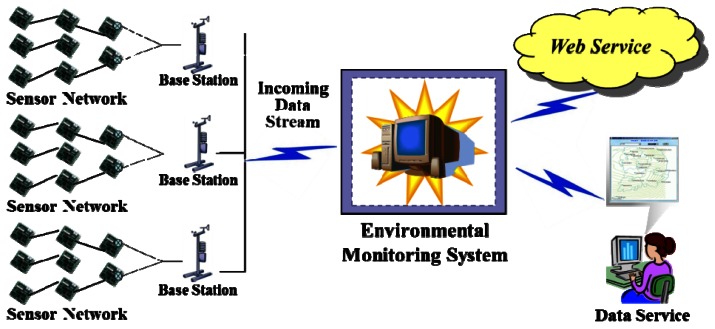
A generic view of applying sensor networks to environmental monitoring.

**Figure 8.**
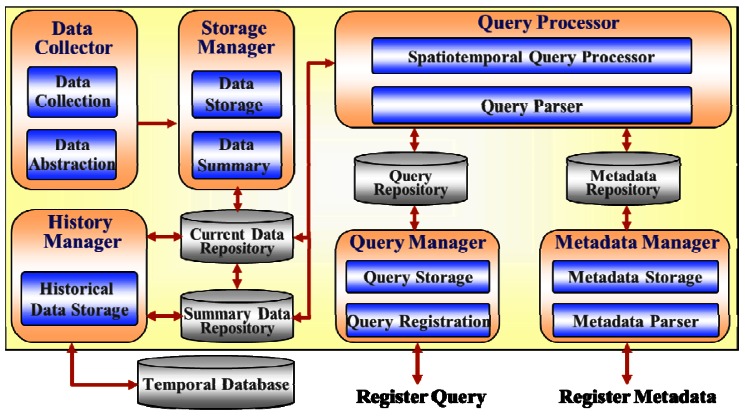
System architecture for environmental monitoring.

Figure 7 depicts the conceptual architecture in which sensor network is deployed to measure information in some region. The sensor network consists of the sensor nodes that collect data according to application-specific requirements and multiple base stations receiving transmitted data. Sensor data sending from base stations are accumulated in the centralized system. They are then organized managed and used for different purposes such as queries or decision making based on some statistical analysis or data mining. Efficient query processing is the goal of this paper. Figure 8 displays the proposed system architecture for processing the incoming data stream.

The detailed functions of the constituents in the system are summarized as follows:

1. *Data Collector*: The data collector gathers the packets of the data stream transmitted by the sensors and converts them into an abstracted sensor data format such as the sensor id, measured value, time, *etc.* The converted data stream is then inserted into the sliding window by the Storage Manager.
2. *Storage Manager*: The storage manager inserts or updates the data stream in the sliding window of the Current Data Repository according to the stream of inserted data values. Expired data in the sliding window are summarized and stored in a Summary Data Repository. It is also stored to disk by the History Manager. Besides, the queries for the analysis and statistical examination of the past data are processed by utilizing the summarized information in the Summary Data Repository.
3. *Query Processor*: The query processor processes continuous queries as well as one-time queries on the data stream. The continuous queries are processed by checking the input data stream with predicates of a list of continuous queries registered in the Query Manager. A spatiotemporal query is also processed by checking the spatial information in the Metadata Repository depending on the query type.
4. *Query Manager*: The query manager can register, update, and delete a continuous query in the Query Repository. Besides, it can extract a list of continuous queries satisfying the query conditions in the query search order of the query processor.
5. *Metadata Manager*: The metadata manager manages the metadata, which is composed of the spatial information and their specifications, *etc.* The locations of the sensors are stored in the Metadata Repository and are utilized for processing spatial and/or temporal queries.
6. *History Manager*: The history manager handles the task of storing expired data in a sliding window to disk.

## 5. Implementation and Running Example

In this study, we implemented a weather monitoring application based on our proposed system architecture. This system monitors the data stream periodically collected from sensors distributed over a wide area and detects any anomalous events. To show the operation of our system, we assume a simple scenario. The temperature sensors distributed in the spatial area collect and transmit the data stream to the server every 2 seconds. The server receives the data from the sensors and monitors them in real-time. The users register the continuous queries that they are interested in for any or all areas. The system performs the registered continuous queries on the incoming data stream according to the query predicates.

**Figure 9.**
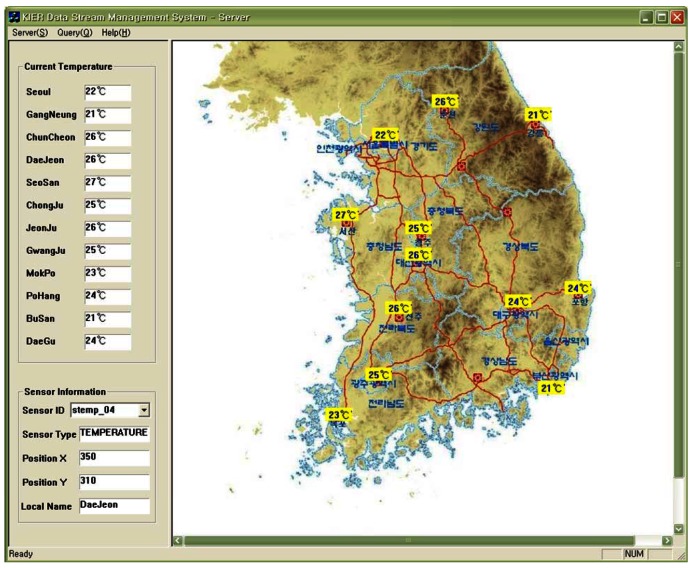
System interface for climate monitoring.

**Figure 10.**
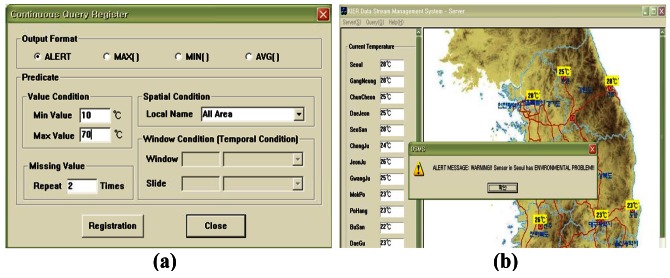
Continuous query registered to detect an environmental event in which (**a**) allows the registering of a query for anomaly detection; (**b**) displays the view of the detected event according to the registered query.

**Figure 11.**
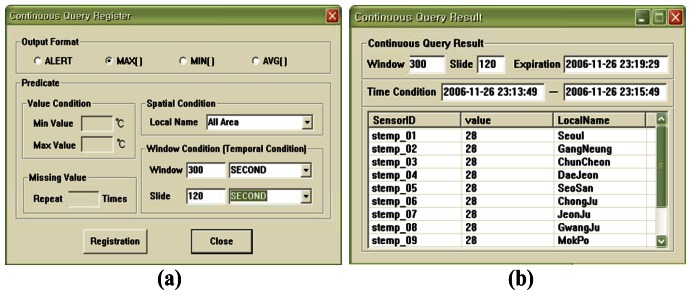
Continuous query registered for spatiotemporal query processing in which (**a**) registers the information for a spatiotemporal query; (**b**) displays the view of the detected event according to the registered query.

Figure 9 shows the interface for the weather monitoring system. The system displays the data collected from the sensors in real-time and then updates the value of the segment or inserts new tuples to represent the changed values after comparing the old values with the latest data in memory. Figure 10 shows the registering of a query to detect anomalous data. The query processor continuously generates the results when the temperature values measured from sensors which are less than 10 degrees or larger than 70 degrees in all areas. The users can register queries that allow them to be informed about any situation. Figure 11 displays the registering of spatiotemporal queries and their results. The user can enter the window range to perform the query continuously. The continuous query is closed when the current time arrives at the end point of the window range.

## 6. Performance Study

We implemented the proposed methods in a weather monitoring system [5]. Our experiments were run on an Intel Pentium 4 2.8 GHz machine, running Microsoft Windows XP, with 1 GB main memory, using Mysql 5.0 and Microsoft Visual C++ 6.0. The dataset consisted of sensor readings collected from 54 sensors deployed in the Intel Berkeley Research lab between 28 February and 5 April 2004 [10]. The dataset contains 2.3 million sensor readings. The Mica2Dot sensors collect the temperature, humidity, light, and voltage values once every 31 seconds.

### 6.1. Experimental Results

In our experiments, we compared the TSI and TPI methods with the NTI method as a naïve approach which does not take account of duplicate data values over time (*i.e.*, sampling based processing by specific time interval). Moreover, we evaluated the performance of these methods with the use of factors such as the number of tuples, average insert time and average query execution time, along with the number of sensor readings obtained from the incoming data stream.

**Figure 12.**
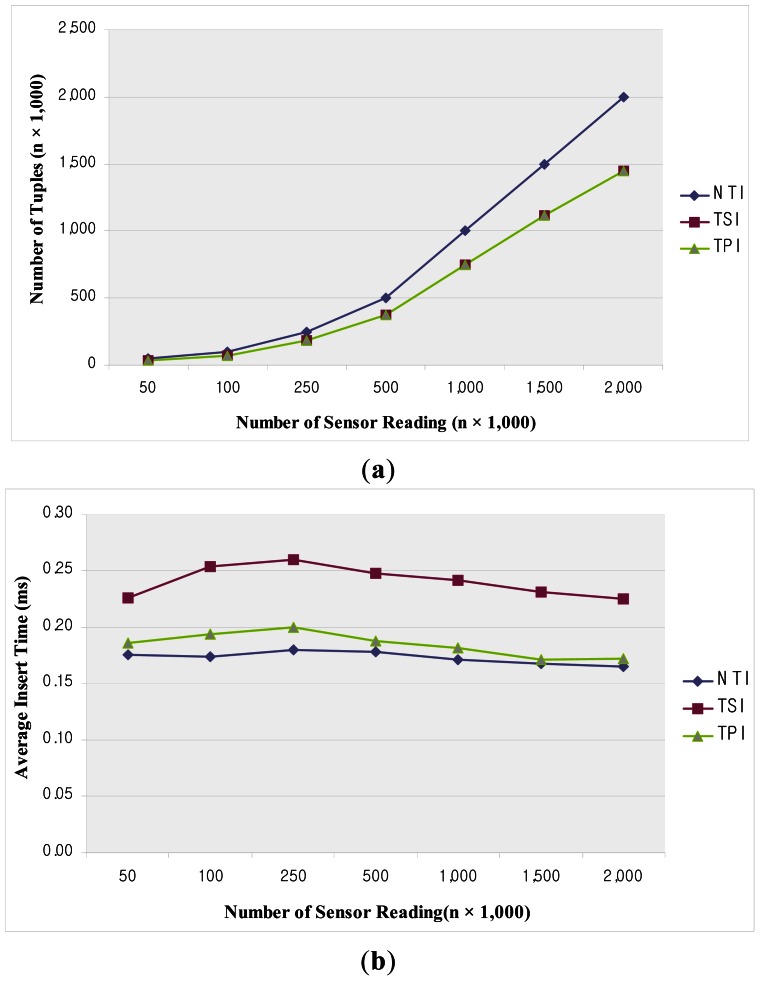
The number of tuples as a function of the number of sensor readings (**a**); and Average insert time as a function of the number of sensor readings (**b**).

Figure 12(a) displays the number of tuples as a function of the number of sensor readings. Since the NTI method inserts all the data values from the incoming stream, the number of tuples stored increases in proportion to the size of the input stream. On the other hand, the TSI and TPI methods discard duplicate values and store the data just once if they do not change over time. Therefore, the number of stored tuples is decreased by about 30%, and both methods give the same number of tuples. Figure 12(b) shows the average insert time as a function of the number of sensor readings

In this experiment, NTI generally outperforms the other algorithms. NTI does not incur any additional cost to store the incoming data stream, because it only conducts insert operations to accomplish this. However, in the case of TSI and TPI, comparisons have to be made between the new and previous data in order to decide whether to store or discard the data values. Therefore, the insertion cost is higher than that of NTI. Especially, since TSI has to conduct update operations as well as insert operations related to the time-point which is stored in the previous timestamp. Its performance is about 25% lower than that of NTI. Besides, TPI does not need any additional update operations, because it stores data stream to a time-point. Since this requires only computation for choosing insertion, its performance is similar to that of NTI. According to this performance result, the update operation is the major reason for the increase in the insertion cost and comparison with the values of the data stream do not much affect the insertion cost. The TSI and TPI methods improve the insertion performance since the number of insertions is reduced due to the discarded data stream.

**Figure 13.**
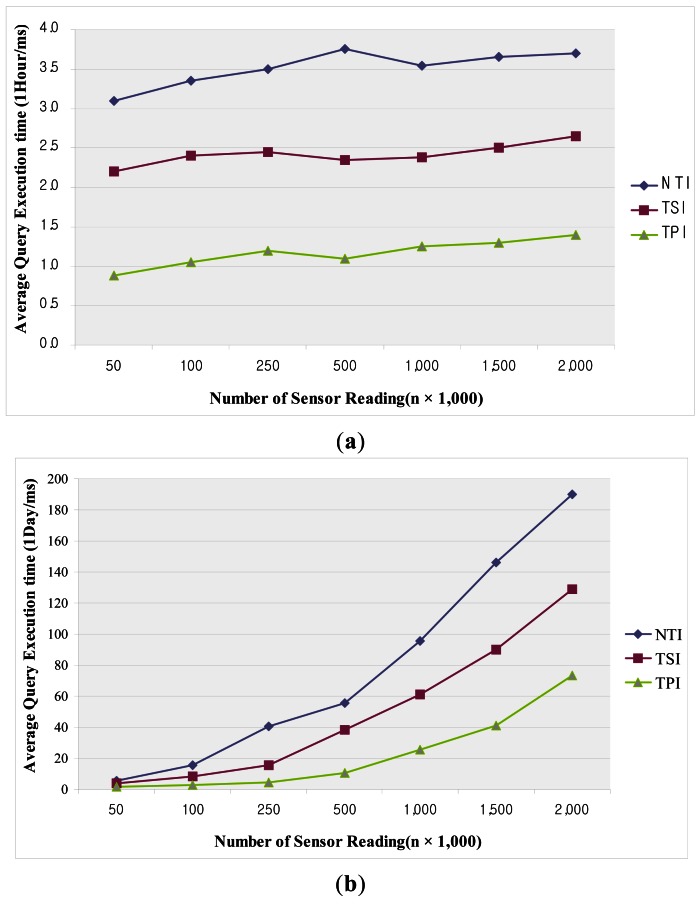
Average query execution time as a function of the number of sensor readings (1 h) (**a**); and (1 day) (**b**).

Figure 13(a) illustrates the average query execution time over a period of one hour as a function of the number of sensor readings. In this experiment, TPI gives the best performance among the various methods. TPI compares only the time-point included in the time-interval of the query predicate, which is a simple operation. Moreover, since the query is performed with a smaller number of tuples than in the case of NTI, fewer operations are required. TSI gives lower performance than TPI. Since TSI requires the time-segments to be compared with the given range query predicate for both the start time and end time, the query operation is rather complicated. NTI gives the lowest performance owing to the large number of operations on tuples required, as compared to the other methods. Figure 13(b) shows the average query execution time over a period of one day as a function of the number of sensor readings. As in Figure 13(b), TPI gives the best performance and NTI the worst. The query processing time is linearly increased with increasing data size. This is due to the number of disk accesses required in the case of historical queries, including a long time-interval.

### 6.2. Discussion

In our experimental results, the performance of TSI and TPI is about 30% better than that of NTI. This performance depends on the variation in the pattern of data collected from the sensor network application. If the data changes slowly, the proposed methods can reduce the storage space required very efficiently, whereas if the data changes dynamically, the performance may be worse due to the operations required for choosing the data to insert.

The insertion cost is the best in the case of NTI, because of its unsophisticated operation. TSI and TPI have disadvantages in terms of their performance due to the insertion operations. These methods handle the stored tuples by using the measured values and temporal attribute, which increases the cost of the operation. Though these methods have the advantage of reducing the number of insertion operations because a number of tuples are discarded before insertion, they cannot reduce the average insertion cost. In most cases, since queries including a comparatively short time-interval are conducted for the tuples in memory, the query cost depends on the number of tuples. In TSI and TPI, the tuples are collected from the sensors, but TSI gives lower performance than TPI because it compares the time-segment from the given query range. Besides, in the case of a query predicate including a long time-interval, the query searches the tuples stored on disk and, therefore, the performance of NTI is very poor, because it has to store many tuples on disk. TSI and TPI have the same cost in terms of the disk access, but for the same short time-interval query the cost becomes different owing to the different comparisons for the time-segment and time-point.

In summary, in most of the experimental results, TSI showed lower performance than TPI. Since TSI represents the input stream in the form of tuples associated with time-segment, it allows the lifetime of the tuples to be handled and all operations involving temporal relations to be supported. In the case of TPI, though it has outstanding performance, since it stores only the time-points, it needs to compute the lifetime of the tuples to support temporal operations.

## 7. Conclusions

A wireless sensor network is a computer network consisting of spatially distributed autonomous devices using sensors to cooperatively monitor physical or environmental conditions, such as the temperature, sound, vibration and pressure at different locations. Wireless sensor networks are now used in many civilian application areas, including environment and habitat monitoring, health care applications, home automation, and traffic control. So far, many studies have been conducted on systems for sensor data stream processing. Nonetheless, they mainly focus on processing continuous queries on the real-time data stream and do not solve the problem of storing the historical data collected from the sensors, which is mandatory for historical queries.

In this study, we introduce a weather monitoring system using the existing temporal and spatial approaches, in order to support spatiotemporal queries, store sensor data. In our system, we have proposed two insertion methods called TSI and TPI for reducing the storage space without any loss of the raw data necessary for queries using the sensors' temporal attributes. In the TSI and TPI methods, we stored the incoming data stream in the form of time-segments and time-points by comparing with the change of the values. Additionally, we proposed a method for reducing the cost of join operations when processing spatiotemporal queries. In particular, the spatial information of the sensors is maintained in memory with the support of a fixed grid when identifying the sensors' locations. By filtering out the list of irrelevant sensors from the query range before making the join operation, its cost is reduced. In our experiments, we compared the TSI and TPI methods with a naïve method called NTI, and the results showed that the proposed methods are better than NTI in terms of the data storage as well as the query execution time. Our ongoing work involves the evaluation of the performance of the proposed methods using the temporal indexing scheme and query processing from multiple sensor data streams.
